# Calamus: A Pen for Microneurosurgical Training

**DOI:** 10.7759/cureus.11428

**Published:** 2020-11-10

**Authors:** Oleg Titov

**Affiliations:** 1 Neurosurgical Training, OPEN BRAIN - Neurosurgical Laboratory of Open Access, Moscow, RUS; 2 Neuro-Oncology, Burdenko Neurosurgical Center, Moscow, RUS

**Keywords:** calamus, invention, microneurosurgery, instrument, training, operative skills, deep and narrow operative field, dexterity, calligraphy, handwriting

## Abstract

Microneurosurgery is Sisyphean labor: dexterity hardly comes and easily goes. The only way to stay ahead is to constantly train. However, it requires a special working place and a lot of expensive equipment, which dramatically decreases the training availability. The author proposes Calamus - a novel tool, which may solve this problem. Calamus is a pen with the anatomy of microneurosurgical instruments. It includes a single bayonet and round handle, a curved tip, and a replaceable refill. Given a lot of similarities between the techniques of microneurosurgery and calligraphy (fine hand movements, working pose, method of controlling the instruments, space management, breathing, psychological state), writing or drawing something using Calamus simulates a crucial neurosurgical skill - working in a deep and narrow operative field (skull base, brainstem, etc.), wherein the quality of technique is objectively reflected by the inky trail. This simple and low-cost instrument can make the microneurosurgical training available for everyone, at every time and place.

## Introduction

High-level microneurosurgical techniques, such as operations on deep brain structures, require hard, long, and unstoppable training [1-3]. However, this is unaffordable for most neurosurgeons due to the necessity of attending a special working place (operative theatre or laboratory) with manifold expensive equipment (microscope, different microinstruments, suture materials, simulators, etc.). Can it be solved?

The answer lies in the field of calligraphy. It is well known that the way of holding and moving most of the surgical instruments is very similar to the technique of writing or drawing by the pen [4-6]. This similarity could provide a unique opportunity for present and future neurosurgeons - to train virtually at any time or place.

However, the usual pen can’t be used for microneurosurgical training due to the following disadvantages: a very low grip area and a short and straight corpus, restricting the view of the pen tip. This article aims to describe a novel writing instrument suitable for everyday microneurosurgical training and to demonstrate the technique for its use.

## Technical report

Calamus

In order to fit the conditions of the deep and narrow operative field, an ideal microneurosurgical instrument must have a precise angled functional tip and a single, bayonet, and round handle [6-8]. The angled tip is better visible than the straight one. The single handle occupies less space than the double one. Bayonet shape allows removing the surgeon’s hands from the view, improving the tip visualization. The round handle provides better rotation compared to a flat or rectangular shape.

Calamus is a writing instrument (specifically, a pen), the anatomy of which satisfies the indicated criteria. Its name derives from the homonymous part of the brainstem - *calamus scriptorius* (“pen nib”) [9].

**Figure 1:**
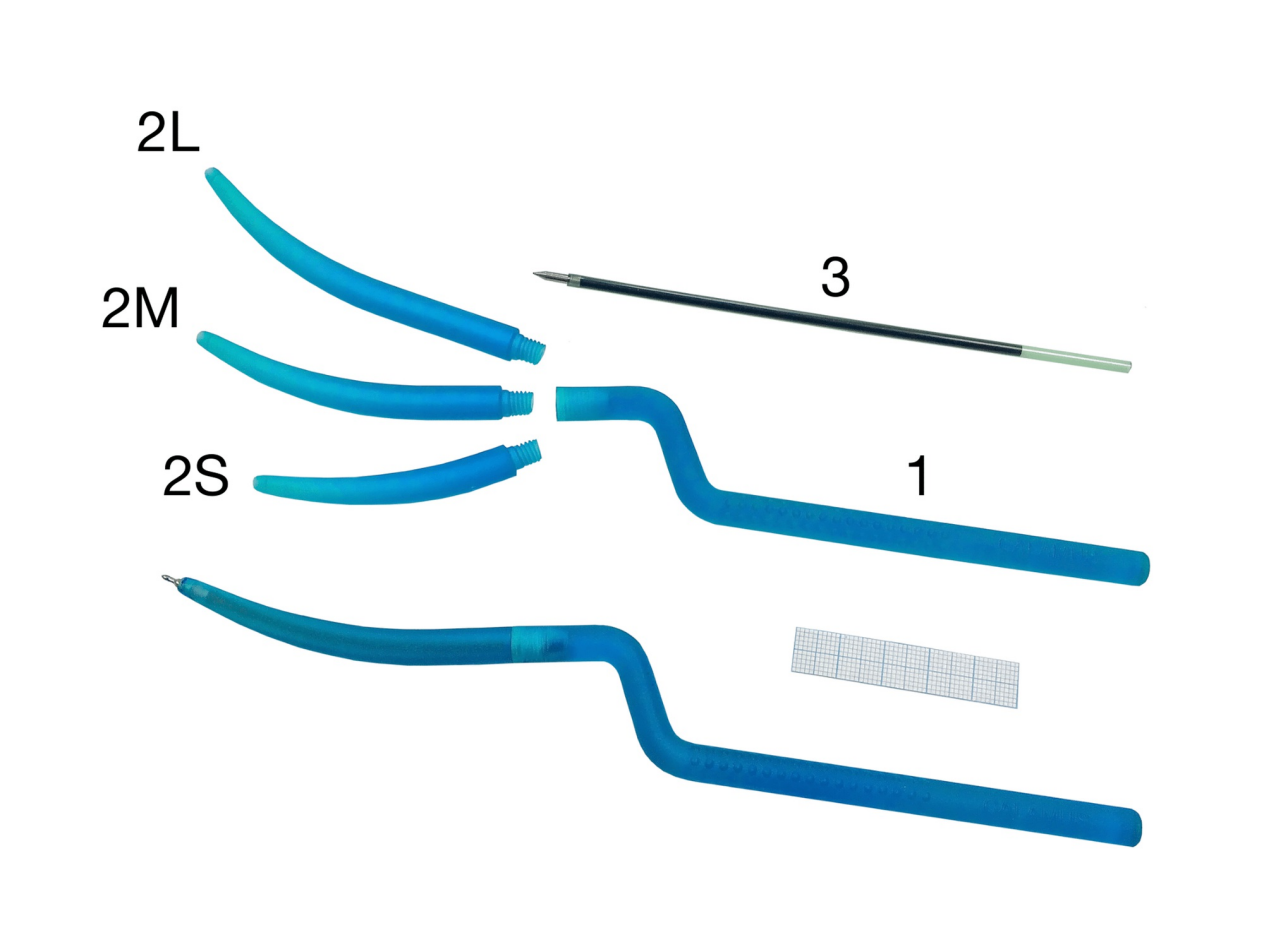
Assembled and disassembled Calamus 1 – handle; 2 – tips (S – small, M – medium, L – large); 3 – refill (can be taken from any usual ballpoint pen and cut with scissors, if necessary) The graph paper indicates the scale.

In the disassembled view (Figure 1), Calamus includes three parts: handle, tip, and refill. The handle is single, bayonet, and round. Its proximal part comprises the grip area, which may be reinforced by many little grooves or protrusions, preventing the slipping. The tip contains a canal for the refill and is made in the form of a cylinder, gradually turning into a more or less curved cone. The refill may be borrowed from any usual ballpoint pen, better with a fine (0.5 mm in diameter), conical or needle-shaped tip. If the refill is longer than the appropriate canal, it can be easily cut with usual scissors.

In the assembled view (Figure 1), Calamus looks like a standard bayonet microneurosurgical instrument, divided into three segments - proximal (holding), intermediate (abducting), and distal (working). The distal segment can have more or less length in order to simulate different grades of the wound depth. For example, 8, 9.5, or 11 cm - superficial, deep, and extra deep according to Rhoton classification [7].

The first samples of Calamus were made from plastic (Tough Photopolymer Resin, Formlabs, USA) using stereolithography.

Ink training

The core idea of “ink training” involves writing or drawing something using Calamus *as small and accurate as possible*, holding and moving it like a real bayonet microneurosurgical instrument. 

The minimal equipment required for the exercises includes the Calamus with an installed refill and a piece of paper.

**Figure 2:**
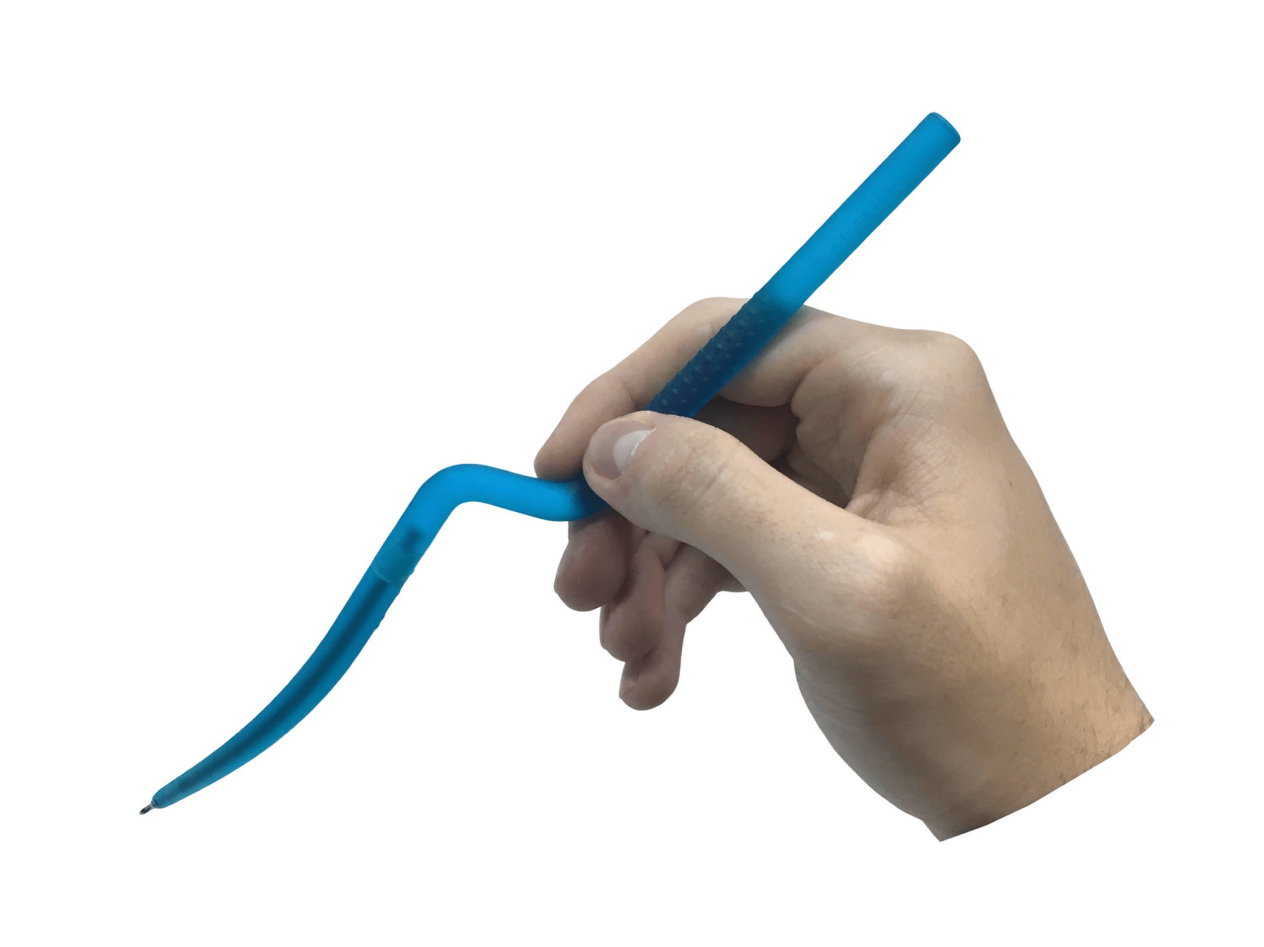
The way of holding Calamus – like a real bayonet microinstrument

In order to hold the Calamus correctly, your hand must create at least four points of fixation around the grip area - three distal and one proximal. The distal points are represented by distal phalanges of the first, second, and third fingers. The proximal point is located on the proximal phalanx of the second finger or its metacarpophalangeal joint (Figure 2).

In order to stabilize the hand, you can use as support the complex of the fifth finger and hypothenar eminence (full brace), only the fifth finger (front brace), only hypothenar (back brace), the tip of the fifth finger (point brace), or you can work without hand support (no brace), depending on the depth of the working field and size of the available stationary surface [10].

For a more realistic representation of the surgical setting, it is advisable to involve a magnification device - loupes or microscope - and a simulator of the deep and narrow operative field.

In the case of the absence of a microscope, the latter can be replaced by a smartphone with an installed magnifier app, fixed using a phone holder. This low-cost 2D-system allows developing eye-screen-hand coordination, which is paramount in endoscopic operations.

**Figure 3:**
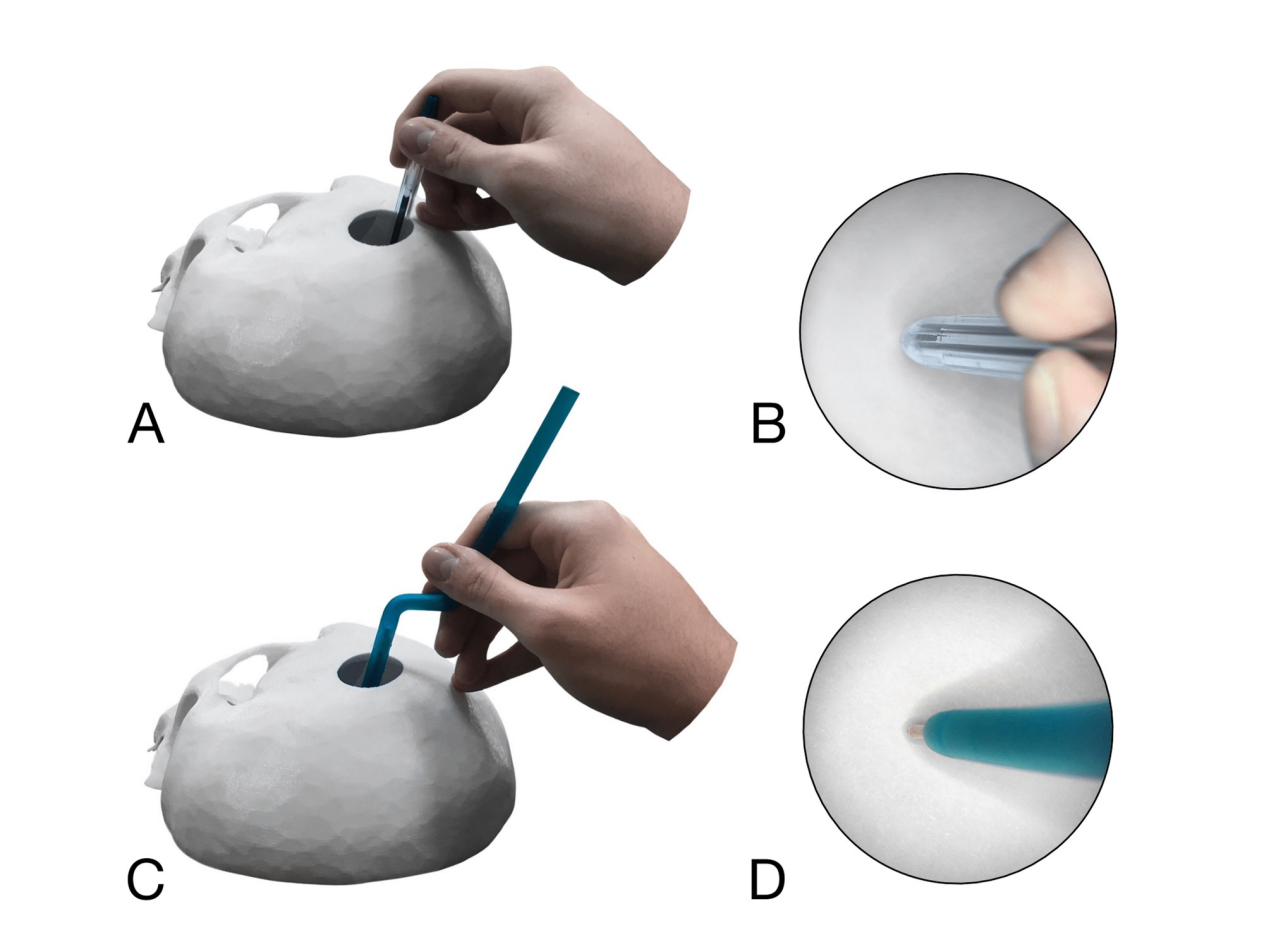
Simulation of working through a keyhole approach (3 cm in diameter), using a usual pen and Calamus (A) Usual pen, macroscopic view; (B) Same, microscopic view; (C) Calamus, macroscopic view; (D) Same, microscopic view. Note that the construction of the usual pen doesn’t let to see the refill tip and comfortably fix the handle, unlike the Calamus.

The deep and narrow operative field can be modeled by an inverted disposable cup with a hole in its bottom. Also, half of a 3D-printed human skull with a “keyhole” can be used (Figure 3).

Moving the Calamus starts from a point touch of its tip to the paper. After that, the latter turns into a canvas for the creation of every conceivable image.

The working hand movements must be limited mainly by motions of the intrinsic hand muscles. In order to fulfill this condition, you should write or draw on a small area that does not allow to involve the wrist or forearm.

**Figure 4:**
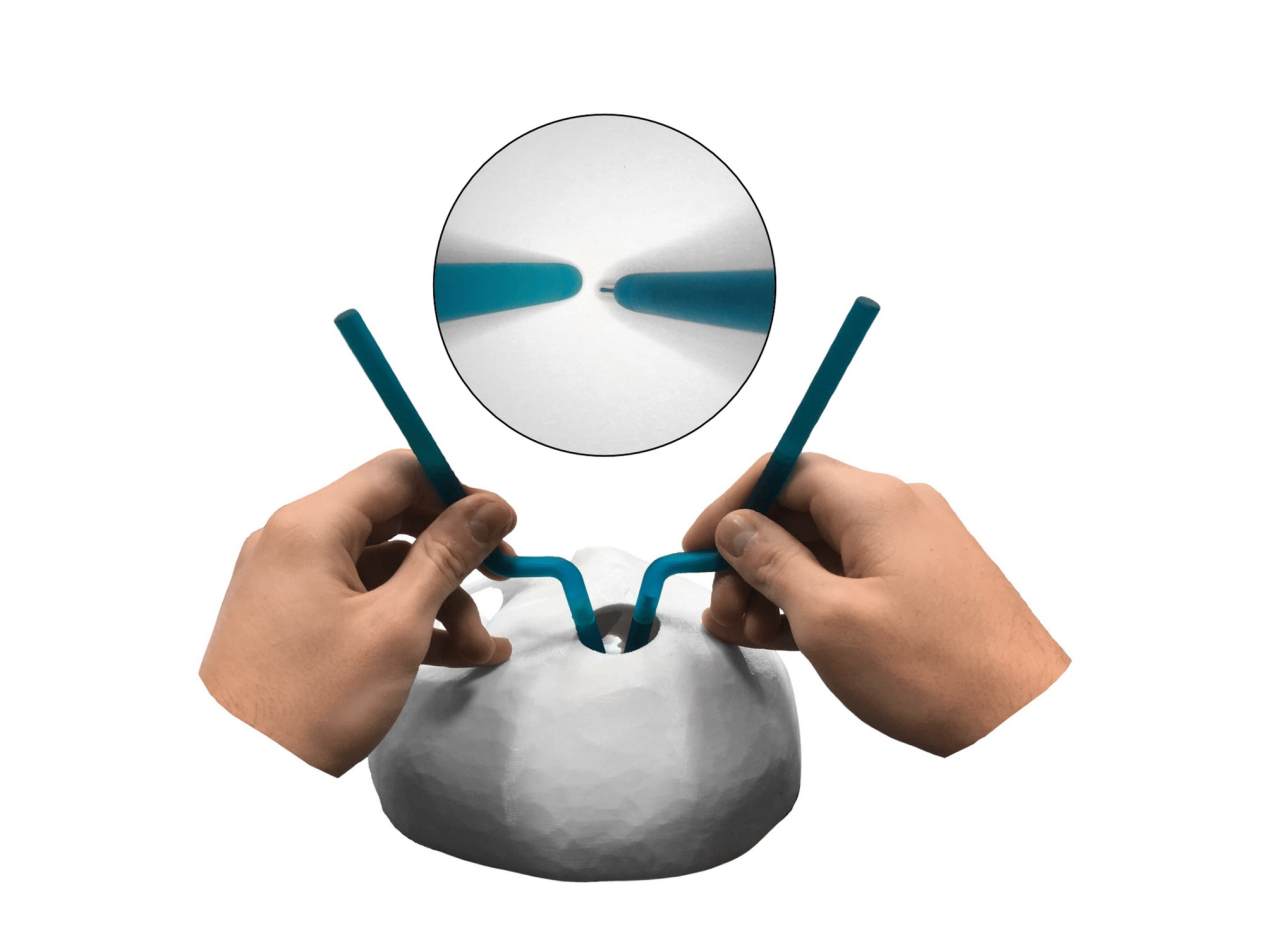
Bimanual “suction-cautery” technique (macro- and microscopic views) The left Calamus without refill plays the role of a suction tube, dynamically fixing and moving the paper. The right Calamus plays the role of a cautery.

It is also advisable to fix the paper dynamically and gradually move it as it fills, using the second Calamus without refill, like a suction tube (Figure 4). This is a partial reflection of the suction-cautery technique [4].

**Figure 5:**
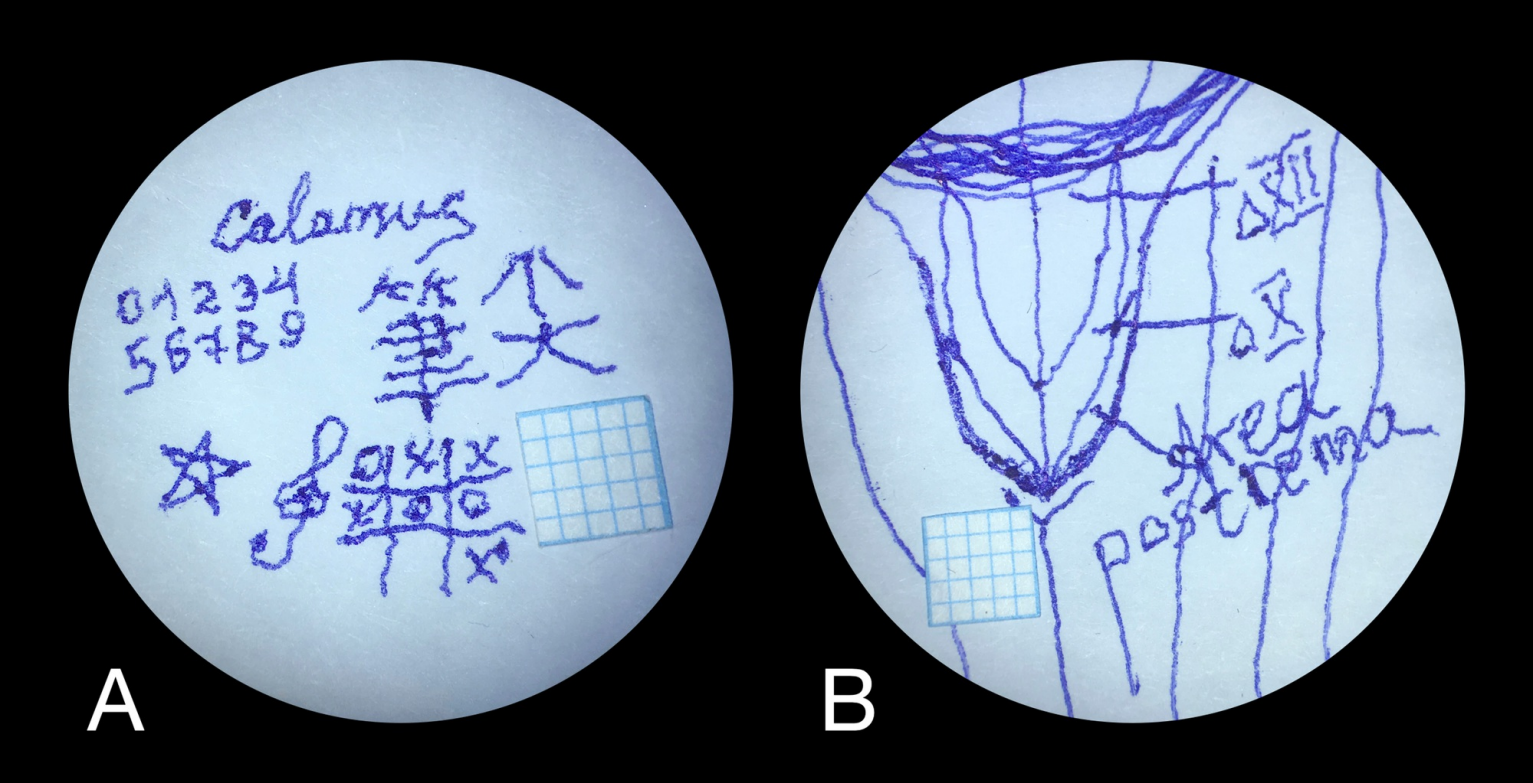
Exemplary “ink training” exercises, made by the author using Calamus, microscope and simulator of the deep and narrow operative field (A) Word “Calamus”; numbers from 0 to 9; Chinese character “筆尖” (meaning “Calamus scriptorius”); star; treble clef; tic-tac-toe. (B) Anatomical sketch of calamus scriptorius, with designations – hypoglossal triangle, vagal triangle, area postrema. The graph papers indicate the picture scale.

Anything can be written or drawn (Figure 5). Points, lines, simple figures, letters, numbers, musical notes, etc. I especially recommend writing oriental characters since they include multiple compounds (strokes), which requires very accurate tip execution [11]. Likewise, you can try some classic western calligraphy exercises [12-14] or draw neuroanatomical sketches. In order to involve an assistant, it is possible to use Calamus in paper-and-pen games (Tic-tac-toe, Sprouts, etc.).

The paper can be inclined, folded, rolled, or crumpled to acquire skills of working with three-dimensional objects. Also, some other kind of 3D writing surface can be used (e.g., papier-mâché).

Ultimately, no matter what is written or drawn, it matters how it is done. This process requires a comfortable body posture and a quiet mind. One has to be calm and able to concentrate on every tip motion. The tip must be clearly and constantly visible. The breathing is also important - write with controlled and continuous exhalation, or in a pause before the next inhalation, when the diaphragm is relaxed and stable. Do not scribble! Treat the paper like a rhomboid fossa: erroneous movement leads to disaster.

“Write your best, or not at all” [13].

## Discussion

A fine line between microneurosurgery and calligraphy

Microneurosurgery is an extremely challenging job, which is built on highly sophisticated psychomotor skills. The burning issue concerning these skills is that they are hard to acquire and easy to lose.

Some studies show that attaining an expert level of neurosurgical performance requires about 10,000 hours of deliberate practice [1]. This path will take “only” 6.9 years if to train five hours per day, six days per week, and 48 weeks per year!

On the other hand, if an already experienced neurosurgeon stops the activity for even a month, the quality of his or her microsuturing technique worsens from 100% to 81%, and for a quarter - up to 56% [2]. Just one break (vacation, sick or maternity leave, etc.) may erode the skill and jeopardize the patients! Therefore, microneurosurgical training has to be long enough, constant, and, ideally, independent of a special working environment (operative theatre, laboratory, and so on).

In this preliminary report, I present Calamus - a novel instrument for microneurosurgical training and technique for its use that may solve this problem and be extensively used in daily life. The idea of Calamus is based on the similarity between calligraphy and microsurgery, which deserves a thorough review. 

Both techniques represent a set of complex psychomotor skills involving fine hand movements [6, 10-14]. Almost the same principle of control of the appropriate instruments is a well-known fact that is reflected in surgical terms such as “writing position,” “pen grip”, or “pencil grip” [5-7].

The working posture is also similar: sitting position, wherein the body is parallel to the table front, the feet are planted equally on the floor, the elbows and, if possible, wrists are freely placed on the table or armrest, the shoulders are relaxed, the neck and back are comfortably straight [5, 12, 14, 15]. Some surgeons prefer to work standing, the pose also existing in calligraphy [4, 14].

Another similarity is the space management: maintenance of correct spacing, alignment, and density of letters and stitches - crucial “working elements” in calligraphy and microsurgery, respectively [10, 12-14, 16].

Finally, one can note the common psychology, which implies staying in a special state of “relaxed concentration” [10, 11, 17]. This state is rarely discussed in the surgical literature, but in the philosophy of Zen Buddhism, tightly linked with oriental calligraphy, it is a crucial concept, known under the name of *mushin*, which can be defined as an open and clear mind, totally focused on every stage of the given task, free from irrelevant thoughts, emotions and other distractions (“mind without mind”) [18-19]. 

However, despite the aforementioned similarities, calligraphy and neurosurgery are still different. One of the main differences consists of the structure of the operative field. For a penman, it is superficial and wide. For a neurosurgeon, it is deep and narrow. Hence the disparity between appropriate instruments and techniques.

In a common way of holding the pen, the distance between its tip and the fingertips is about 2-3 cm, while in neurosurgery, this distance may exceed 15 cm [7, 12-14]. Not every pen has such a length, so, even if one takes it close to its proximal end, it will be impossible to provide stable fixation (Figure 3A).

The most comfortable pen inclination concerning the paper is usually about 50° [12-14]. However, a ballpoint pen can be inclined up to 35° until stopping the ink outflow (this can be measured using a protractor). Therefore, the “angle of attack” in calligraphy is approximately 110°. In neurosurgical approaches, this parameter can be much more limited. For example, axial and sagittal angles of attack during microscopic sublabial approach to the pituitary gland are respectively 14.7 ± 1.3° and 14.9 ± 1.9° [20]. It corresponds to almost vertical pen position (so-called “brush grip” [11]), in which the tip can’t be seen from above since it is closed by the boundaries of the operative field, the straight pen corpus, and the writer’s hand (Figure 3B).

Besides, the brace in calligraphy is usually full, so, given the low grip position, the problem of tremor is not as vital as in neurosurgery. Therefore, regular pen and technique for its use are unsuitable for training.

Advantages

Calamus is designed to correct the aforementioned deficiencies and meet the needs of microneurosurgeons for a reliable and affordable training tool. It has the following advantages. 

First, it reflects the features of real instruments: the handle is single (more compact), bayonet (provides uncluttered view), and round (easy to rotate). The tip is angled (clearly visible). This allows working in a deep and narrow field.

Second, the high grip area strongly complicates the tip control, enabling one to experience and train different ways of tremor suppression: adjustment of posture, hand position, breathing, and mood. Given that there is no support from the walls of the “surgical corridor”, the ink exercises might be even somewhat harder than the real operative technique, which makes this training method challenging and thus helpful even for an expert.

Third, and most importantly, it leaves an inky trail. So, it serves as a motion sensor, which reflects the quality of technique and allows monitoring the visuomotor coordination. This feature can theoretically be used for objective assessment of microneurosurgical abilities and aptitudes.

Fourth, it is very undemanding. Usually, the training requires a lot of instruments with different functions. Calamus may replace them all. The paper plays the role of expensive biomaterial (e.g., cadaver head). Also, there is no need for suture materials and a special working place. Everyone can train everywhere, anytime.

Limitations

Despite the outlined benefits, it is worth noting several limitations of Calamus. 

First, its current design includes a single-shaft handle (like dissectors, hooks, curettes, diamond knives, etc.), which doesn’t allow to perform the movements of squeezing and spreading, unlike double-shaft instruments (forceps, scissors, needle holders, etc.). Anyway, it doesn’t significantly ease the distal end execution.

Second, the round handle and angled tip represent only one option of the structure of microinstruments. Not all neurosurgeons prefer this kind of construction, and some of them may find it less comfortable than flat-type tools with straight tips. However, in the future, Calamus may be adjusted to the diversity of personal preferences.

Third, this version of Calamus is made from plastic, unlike standard microinstruments, usually consisting of metal (stainless steel, titanium, etc.). It may slightly decrease the realism of “ink training”, but this minor issue can also be resolved.

## Conclusions

Calamus is a new, simple and low-cost instrument for microneurosurgical training that unites technologies of calligraphy and microneurosurgery and allows to perfect delicate skills of working in the deep and narrow operative field, at any time and place. This tool may help young and future neurosurgeons (residents and students), who have to master microsurgical skills, and the experts, who have to sustain them. Further studies are needed to verify its validity.

## References

[1] Omahen DA (2009). The 10,000-hour rule and residency training. CMAJ.

[2] Cokluk C, Aydin K (2007). Maintaining microneurosurgical ability via staying active in microneurosurgery. Minim Invasive Neurosurg.

[3] Belykh E, Byvaltsev V (2014). Off-the-job microsurgical training on dry models: Siberian experience. World Neurosurg.

[4] Hernesniemi J, Niemelä M, Karatas A (2005). Some collected principles of microneurosurgery: simple and fast, while preserving normal anatomy: a review. Surg Neurol.

[5] Stangel JJ, Lahr DY (1984). Microsurgery, Microinstruments, and Microsutures. Handbook of Microsurgery.

[6] Yadav YR, Parihar V, Ratre S, Kher Y, Iqbal M (2016). Microneurosurgical skills training. J Neurol Surg A Cent Eur Neurosurg.

[7] Rhoton AL Jr (2003). Operative techniques and instrumentation for neurosurgery. Neurosurgery.

[8] Matsumura N (2012). A new bayonet spring microsurgical instrument handle with a bar for microneurosurgery. Surg Neurol Int.

[9] Olry R, Haines DE (2009). The pen nib and the bolt: the rhomboid fossa of the fourth ventricle or the symbol of the censorship of the press?. J Hist Neurosci.

[10] Lawton MT (2018). Seven bypasses: tenets and techniques for revascularization.

[11] Li W (2009). Chinese writing and calligraphy.

[12] Knowles M ( 1881). Real pen work: self-instructor in penmanship. Knowles & Maxim, Pittsfield, Massachusetts 1881.

[13] Gaskell GA (1884). Gaskell's guide to writing, pen-flourishing, lettering, business letter-writing, etc. New York 1884.

[14] Spencer RC, Spencer HC, Spencer PR, Spencer HA, Spencer LP (1879). New Spencerian compendium of penmanship. https://archive.org/details/new-spencerian-compendium-of-penmanship-high-resolution/mode/2up.

[15] Acland RD (1989). Practice manual for microvascular surgery.

[16] Lahiri A, Sebastin SJ, Yusoff SK, Sze Chong AK (2016). Computer aided assessment in microsurgical training. J Hand Surg Asian Pac Vol.

[17] Xu M, Kao HS, Zhang M, Lam SP, Wang W (2013). Cognitive-neural effects of brush writing of Chinese characters: cortical excitation of theta rhythm. Evid Based Complement Alternat Med.

[18] Yagnick NS, Deora H, Tripathi M, Mohindra S, Batish A (2019). Zen and the art of neurosurgery: thought becomes action. World Neurosurg.

[19] Hashi H (2016). The significance of “mushin”: the essential mind of Zen Buddhist philosophy for humans in a contemporary world. Asian Studies.

[20] Elhadi AM, Hardesty DA, Zaidi HA (2015). Evaluation of surgical freedom for microscopic and endoscopic transsphenoidal approaches to the sella. Oper Neurosurg.

